# Tracking Fish Lifetime Exposure to Mercury Using Eye Lenses

**DOI:** 10.1021/acs.estlett.2c00755

**Published:** 2022-12-14

**Authors:** Hadis Miraly, N. Roxanna Razavi, Annabelle A. Vogl, Richard T. Kraus, Ann Marie Gorman, Karin E. Limburg

**Affiliations:** † State University of New York College of Environmental Science and Forestry, Syracuse, New York13210, United States; ‡ U.S. Geological Survey, Great Lakes Science Center, Lake Erie Biological Station, 380 Huron Street, Huron, Ohio44839, United States; § Fairport Fish Research Station, Ohio Department of Natural Resources, 1190 High Street, Fairport Harbor, Ohio44077, United States; ∥ Department of Aquatic Resources, Swedish University of Agricultural Sciences, SE-750 07Uppsala, Sweden

**Keywords:** otoliths, round goby, diet, Lake Erie, St. Lawrence River, Baltic Sea, hypoxia

## Abstract

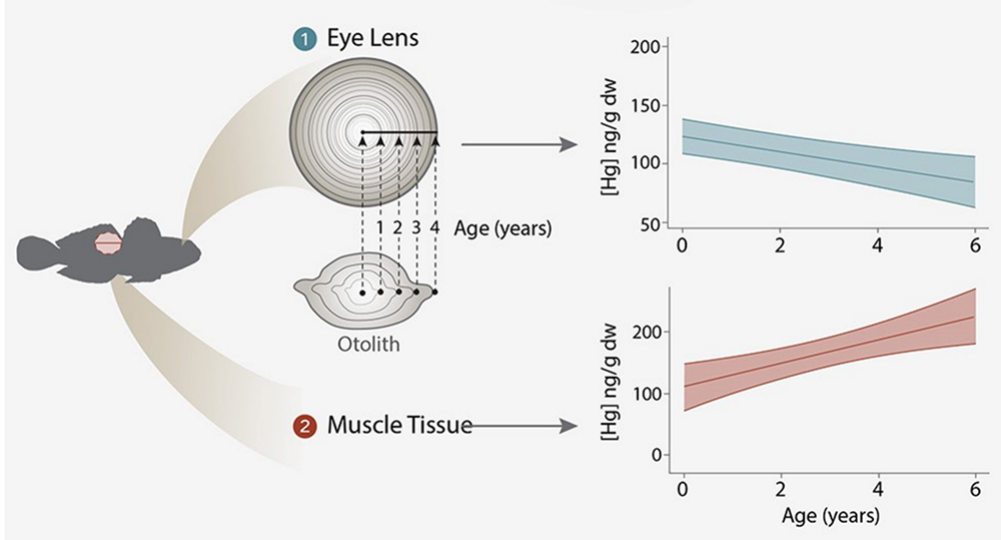

Mercury (Hg) uptake in fish is affected by diet, growth, and environmental factors such as primary productivity or oxygen regimes. Traditionally, fish Hg exposure is assessed using muscle tissue or whole fish, reflecting both loss and uptake processes that result in Hg bioaccumulation over entire lifetimes. Tracking changes in Hg exposure of an individual fish chronologically throughout its lifetime can provide novel insights into the processes that affect Hg bioaccumulation. Here we use eye lenses to determine Hg uptake at an annual scale for individual fish. We assess the widely distributed benthic round goby (*Neogobius melanostomus*) from the Baltic Sea, Lake Erie, and the St. Lawrence River. We aged layers of the eye lens using proportional relationships between otolith length at age and eye lens radius for each individual fish. Mercury concentrations were quantified using laser ablation inductively coupled plasma mass spectrometry. The eye lens Hg content revealed that Hg exposure increased with age in Lake Erie and the Baltic Sea but decreased with age in the St. Lawrence River, a trend not detected using muscle tissues. This novel methodology for measuring Hg concentration over time with eye lens chronology holds promise for quantifying how global change processes like increasing hypoxia affect the exposure of fish to Hg.

## Introduction

Understanding exposure of fish to mercury (Hg), a pollutant of global concern, is important to protect fish health as well as their consumers, both wildlife and humans, from the neurotoxic effects of methylmercury (MeHg).^1^ Fish obtain >90% of Hg through diet; thus, species and trophic position are strong determinants of fish Hg concentrations ([Hg]).^2^ Ontogenetic shifts and/or the introduction or loss of a prey item can have important consequences for Hg bioaccumulation.^3,4^ Age and fish growth rates also influence [Hg].^5,6^ Further, environmental conditions influence the bioavailability of Hg to fish. For instance, hypoxia increases the methylation activity of bacteria that produce MeHg.^7,8^ Thus, numerous factors must be considered when assessing fish Hg uptake.^2,9^

There is currently no method for reconstructing the chronology of exposure of fishes to Hg over a lifetime. Research and monitoring most often use muscle tissues or whole fish to assess Hg exposure, and while beneficial for inferring human and ecological risk, this approach has inherent limitations. Changes in protein/lipid content affect [Hg] in muscle tissues within and/or among species and create problems for temporal or spatial comparisons.^10,11^ Large sample sizes across a range of fish lengths are needed to capture ontogenetic shifts,^12^ seasonal variations,^13^ or rare “pulse” events^14^ that could increase exposure over a short period of time. More importantly, muscle sampling and whole body sampling represent cumulative lifetime exposure, with elimination rates that vary by species and sex.^9^ A method that tracks temporal dynamics, especially at the level of the individual, would aid in assessing life history or environmental drivers of Hg exposure.

Interest in eye lenses as archives to document migration, diet, habitat,^15−19^ and contaminant exposure^20,21^ has recently spiked. As with otoliths, lenses are incrementally grown from the embryonic stage and are inert.^22^ However, they are organic proteinaceous tissues and bind ions such as rubidium (Rb), copper (Cu), and Hg with the sulfhydryl groups of proteins.^14^ The outer cortex is composed of live cells that are eventually overgrown, lose their organelles, and become transparent.^14,22^ Importantly, they are metabolically stable and thus provide a life-long record of elements and isotopes.^14,15,22^ Chemical reconstruction at a specific time point is possible because of the linear allometry between the lens radius and fish body size for a given species.^22^ In the case of Hg, Korbas et al. identified that the eye lens cells preferentially accumulate MeHg compared to other body parts,^23^ a finding confirmed for wild fish.^21^

These characteristics make eye lenses good structures for providing chronological records of Hg exposures throughout a fish’s lifetime.^23,24^ Early work found that fish eye lenses showed location specific [Hg] that correlated with anthropogenic Hg inputs, demonstrating that [Hg] in lenses can act as an indicator of Hg bioavailability.^14,24^ Whole eye lenses confirmed differential Hg bioavailability in field studies of damselfish (*Parma microlepis*)^14^ and golden gray mullet (*Liza aurata*).^21^ To the best of our knowledge, only Dove^24^ attempted to characterize Hg in early versus late life stage using eye lens [Hg] but did not reconstruct continuous lifetime exposure.

Here, we propose a novel technique that combines the use of otoliths, a standard structure used to age fish, with Hg lens chemistry to reconstruct chronologies of Hg exposure in individual fish. As otoliths and eye lenses both grow in proportion to body size, otolith annuli are used to parse eye lens Hg transects into mean annual concentrations. We hypothesized that eye lenses would provide novel insights into differences in Hg bioaccumulation across aquatic ecosystems, as eye lenses would show greater fidelity for reconstructing past exposure compared to traditional techniques.

## Materials and Methods

### Study Species and Sites

The round goby (*Neogobius melanostomus*), native to the Ponto-Caspian region, was introduced into European and North American waters^25,26^ through ballast water in cargo ships and has become one of the most abundant invasive species in both continents.^25,26^ Round goby was used as a model species for this study because populations reside in many geographically distinct areas, can adapt to varying physical and chemical conditions, and have a wide range of diets, so they can thrive in different ecosystems.^26^ The round goby is a benthic species in close contact with sediments that are Hg sinks and is relatively tolerant to hypoxia.^27^ Round goby specimens from the Baltic Sea (*n* = 28), Lake Erie (*n* = 71), and the St. Lawrence River archipelago draining Lake Ontario (*n* = 28) were chosen to represent a broad variation in ecosystem types and provide a comparison to Hg life histories of round goby among different invaded ecosystems. Individuals were pooled among sites with the assumption that diets were more similar within than among ecosystems; known differences in hypoxia exposure between sites for the Baltic Sea and Lake Erie will be the focus of a future study. The primary sources of Hg in Lake Erie and the St. Lawrence River are watershed-derived and industrial,^28^ and in the Baltic Sea, Hg derives from atmospheric, watershed, and industrial sources.^29^ Among our study ecosystems, the Baltic Sea is most severely impacted by hypoxia and increasingly experiencing anoxia.^30^ Lake Erie’s central basin frequently experiences hypoxia, while the western basin experiences it less frequently and episodically.^31−33^ We expect that round goby specimens from the St. Lawrence River have lower hypoxia exposure compared to those from the Baltic Sea and Lake Erie. Details about fish age and length ranges and fish sampling and processing can be found in the Supporting Information.

### Chemical Analyses

A Direct Mercury Analyzer using atomic absorption spectrophotometry (DMA-80, Direct Mercury Analyzer, Milestone SRL) was used to measure the total Hg content (nanograms per gram) of approximately 0.01–0.02 g of each freeze-dried, homogenized fish sample. Muscle tissues were analyzed for all Baltic Sea samples and nearly all St. Lawrence River fish [except four of the smallest individuals that were analyzed as whole fish, with eyes removed (Figure S1)]. Whole fish were homogenized for all Lake Erie fish. To convert whole fish to muscle tissue equivalents, we applied the regression equation in Peterson et al.^34^ often used to convert between tissue types.^35^ Details about quality control and quality assurance are provided in the Supporting Information.

Air-dried eye lenses were analyzed for Hg concentrations using laser ablation inductively coupled plasma mass spectrometry (LA-ICPMS) at the Analytical and Technical Services Laboratory at SUNY-ESF (Syracuse, NY). While ^199^Hg and ^200^Hg can be measured, we used the ^202^Hg concentrations to generate total Hg concentrations using an external calibration. Our conversion equation is as follows:
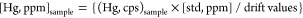
where cps is counts per second, std is the total [Hg] from the certified reference material DORM-4 (fish protein homogenate, National Research Council Canada, 0.412 ± 0.036 ppm), and drift values were calculated from cps interpolated between standard measurements, taken and the start and end of the run, as well as hourly to correct for instrument drift. A 193 nm Teledyne CETAC Analyte Excite Excimer Laser Ablation System was coupled to an iCAP TQ ICPMS instrument to ablate solid material from the epoxy-embedded and polished eye lens cross sections on petrographic glass slides. Preablation conditions were set to 10% power, 135 μm track width (spot size), 50 μm/s scan speed, and 0.9 j/cm^2^ fluence to remove surface contamination along the transect, which was conducted prior to the collection of data. The ablation parameters were set to 10% power, 110 μm spot size, 4 μm/s scan speed, and 0.9 j/cm^2^ fluence. Details about the external calibration method are provided in the Supporting Information.

### Data Analysis

#### Age Estimation and Chemistry of Eye Lenses

**Figure 1:**
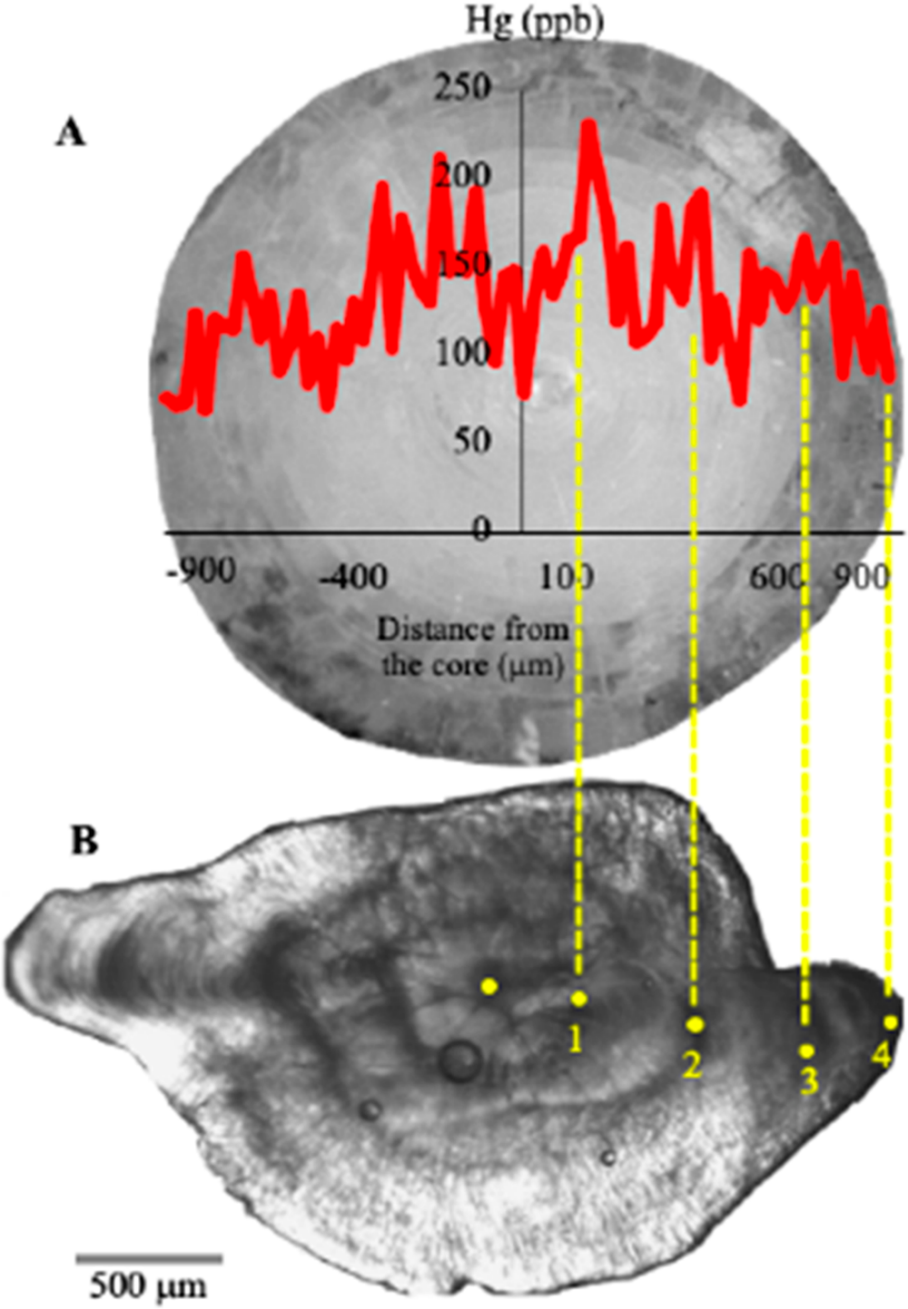
(A) Example of a round goby eye lens Hg profile and (B) corresponding otolith. Otolith lengths at age were measured as the percentage distance along the otolith radius. Corresponding eye lens radii are normalized proportionally to otolith annuli to produce eye lens radii at age. Then using the eye lens radius at age and Hg profile on the laser transect line, the eye lens average [Hg] per year of life of each individual fish was estimated.

To estimate the location of the growth layer in eye lenses, the radial distance from the eye lens core to the outer edge was normalized to the corresponding axial distance along the otolith total length [core to the edge (Figure S2)]. Otolith annuli were quantified as the percentage distance along the otolith axis (otolith length at age); those percentages were then applied to the eye lens transect (Figure 1). The average [Hg] by age was calculated using the average [Hg] that was proportionally related to the otolith annuli, which can incorporate one or more lens layers. Note that we did not measure Hg in the individual layers but across a transect of the eye lens. Because of overall radial symmetry in elemental^36^ and specifically Hg profiles (Figure S3), the core to one edge was used to calculate Hg concentrations. Proportional averages corresponding to otolith annuli from the core to the outer edge of the lens are reported. Graphs displaying individual level variation by ecosystem are provided in the Supporting Information (Figure S3). We found relative stability in profiles of sulfur (a major constituent of proteins) and similar trends among ecosystems, suggesting that there is stability in the biochemical composition across the eye lens.

#### Statistical Analysis

To examine the assumption of isometric growth in otoliths and eye lenses, we assessed the strength of the relationship between otolith length and eye lens radius using Pearson’s correlation analysis. To examine the differences in [Hg] in eye lenses among different ages and ecosystems, a linear mixed-effects regression model (LME) was used.^37^ The annual [Hg] in eye lenses was used as the response variable; the ecosystem and age were used as a fixed effect, and an individual fish was used as a random effect. Intercepts and slopes were allowed to vary by individual. Pairwise comparisons were estimated using marginal means to interpret the interaction effects between ecosystems and age groups. Interaction plots of eye lens average [Hg] by age for each ecosystem from the model were generated. Because there was only one measurement per individual (no random effect of the individual), linear regressions were used to assess the age effect on muscle tissue [Hg] for each ecosystem.

Finally, we wanted to test if recent exposure (as assessed via muscle tissues) and the most recent growth year of eye lens provided the same estimate of Hg exposure. We tested for a relationship between muscle tissue [Hg] and final year eye lens [Hg], as well as among different ages and ecosystems, using a three-way analysis of variance (ANOVA). Age, ecosystem, and tissue type were treated as independent categorical variables, and [Hg] was treated as the dependent variable in the analyses. Tukey post hoc tests were used for multiple comparisons of means. To test the relationship between muscle tissue [Hg] and fish total length (millimeters) for each ecosystem, a simple linear regression model was used (Figure S1). Residuals of all linear models were examined and met assumptions of linearity, normality, and homoscedasticity. Statistical analyses were performed with R programming language version 4.2.0

## Results and Discussion

Eye lenses have been proposed to assess exposure to contaminants (especially Hg and lead),^14,38,23,21,39,40^ and we developed a method for assigning fish [Hg] to eye lens chronology and applied this novel technique to calculate the annual Hg variations in each individual fish. Using this approach, we successfully provided a comparison of Hg fingerprints of round goby from three ecosystems. The cumulative values of the observed average ± standard deviation (SD) of [Hg] in eye lenses (i.e., from the core to the edge) of Baltic Sea, Lake Erie, and St. Lawrence River individuals were 75 ± 40, 85 ± 38, and 115 ± 32 ng/g of dry weight (dw), respectively, while the values of the average ± SD [Hg] of muscle tissue samples were 131 ± 64, 156 ± 52, and 164 ± 94 ng/g of dw, respectively. There was a strong significant Pearson correlation (*r* = 0.82; *p* < 0.05) between eye lens radius and otolith length (core to edge) when fish were combined from the Baltic Sea (*n* = 28), Lake Erie (*n* = 60), and the St. Lawrence River (*n* = 26) (Figure S2).

### Hg History in Eye Lenses and Muscle Tissue

**Figure 2:**
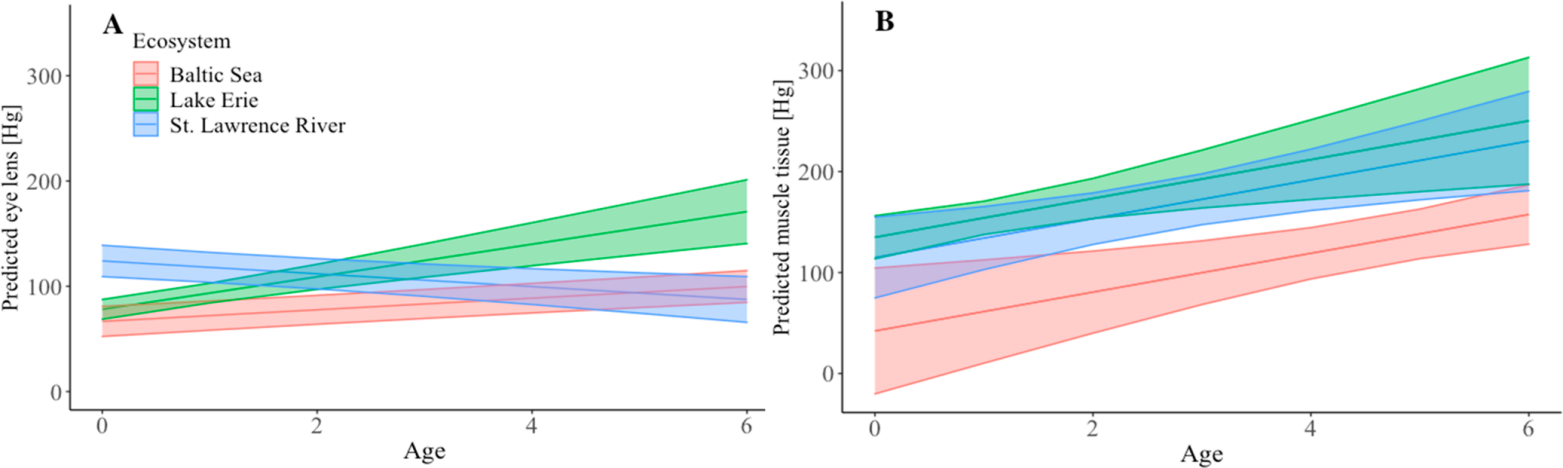
(A) Linear prediction for eye lens average [Hg] (nanograms per gram of dry weight) by age for different locations, based on the fitted linear mixed-effects model. (B) Linear prediction for muscle tissue average [Hg] (nanograms per gram of dry weight) by age for different locations, based on the fitted linear regression model. Shading indicates 95% confidence intervals around the means.

The models predicted a different pattern in eye lense [Hg] and muscle tissue [Hg] by age for the St. Lawrence River (Figure 2). The annual eye lens [Hg] increased with age in the Baltic Sea and Lake Erie but decreased with age in the St. Lawrence River (Figure S3). The mixed-effects model showed a linear association between eye lens annual [Hg] and age modified by ecosystem [interaction effect: *F*_(8,277)_ = 7.559; *p* < 0.0001]. Age-based comparisons showed the St. Lawrence River had a higher eye lens [Hg] for younger fish only (Figure 2 and Figure S4). There was an increasing trend in muscle tissue [Hg] with age in all ecosystems (Figure 2), but the linear regression model showed no difference between muscle tissue [Hg] and age modified by ecosystem [interaction effect: *F*_(6,100)_ = 0.7417; *p* > 0.05].

This technique validated diet shifts described in St. Lawrence River round goby.^41^ Diet is a well-known control on Hg bioaccumulation in fishes. Our finding that eye lens [Hg] in individual St. Lawrence River round goby decreased as fish aged is in agreement with a diet study that showed young St. Lawrence River round goby individuals consume opportunistically but older ones consume a combination of dreissenid mussels and Hydrobiidae, which causes a decline in δ^15^N (a diet proxy).^41^ Because [Hg] is strongly predicted by the δ^15^N of aquatic organisms,^42,43^ it follows that younger round goby individuals are feeding on contaminated prey at a higher trophic level compared to older round goby in the St. Lawrence River. This trend was not detected in muscle tissue [Hg] of the same samples from the St. Lawrence River. This demonstrates that eye lens Hg confirms and provides additional insight compared to traditional diet analyses, without relying on the need to collect fish of a variety of ages, as the eye lens provides a lifetime chronological record of exposure.

In contrast, in the Baltic Sea and Lake Erie, the model predicts that eye lens [Hg] in round goby increases as fish grow older. Round goby specimens from the Baltic Sea and Lake Erie are exposed to hypoxia, which can increase Hg methylation and bioavailability.^7,8,44^ In Lake Erie, despite a considerable decline in sediment [Hg], piscivorous species [Hg] increased after round goby became established.^45^ The round goby as a benthic species may be more vulnerable to hypoxia-induced MeHg bioavailability. Note that the predictions of the model for [Hg] in older round goby diverge in these two ecosystems, with Lake Erie round goby showing higher predicted concentrations at older ages. Older round goby specimens have not been easy to collect in Lake Erie in recent years likely due to declines in the round goby population and in overall forage abundance, combined with historically high numbers of predatory fishes. Additionally, habitat compression from increased levels of hypoxia may result in a greater degree of top-down predatory control. Kraus et al.^46^ showed that more fish aggregate near the edge of hypoxia. In addition to the changing Hg bioavailability, oxygen-depleted areas can reduce benthic communities and increase prey catchability in Lake Erie.^46^ The method presented here confirms that Hg fingerprints of fish in different locations are measurable and comparable using eye lenses. Consequently, lifetime Hg fingerprints hold promise for tracking lifetime diet shifts of fish in different ecosystems; this type of data is highly valuable for identifying species and ecosystems at elevated risk from Hg exposure.

### Eye Lens and Otolith Length Relationship

We found that the eye lens radius was highly correlated with the otolith axial length [*r* = 0.82; *p* < 0.05 (Figure S2)]. Given that otoliths typically deposit visible growth increments proportional to size at age,^47,48^ they can be used to parse eye lens radius into annual growth zones. Our results using otoliths as a proxy of body size are in agreement with those of other studies^19,22^ that found strong linear relationships between body length and eye lens diameter in different fishes. For instance, Quaeck-Davies et al.^22^ examined lifetime isotopic fingerprints using eye lenses. Given that fish age cannot be determined directly by eye lenses, they tested the use of fish length at age to estimate eye lens radius at age. We associated the otolith length at age to eye lens radius at age. We suggest designing an experiment to compare otoliths and eye lens growth rates from the embryonic development stage of fish to the adult stage, to further clarify the growth relationship between eye lenses and otoliths.

### [Hg] in the Final Year’s Growth of Eye Lens Compared with [Hg] in Muscle Tissue

Our analysis demonstrated that, unlike muscle tissue that provides cumulative Hg fingerprints, eye lenses represent a continuous record of [Hg] over a fish’s lifetime. There was no significant difference between muscle tissue [Hg] and final year eye lens [Hg] by age in each ecosystem [interaction effect: *F*_(6, 200)_ = 1.102; *p* = 0.3], except for St. Lawrence River age 3 [*p* = 0.01 (Figure S5)]. Although not significant, fish muscle tissue [Hg] and final year of eye lens [Hg] diverged for older fish, with an increasing [Hg] observed in muscle tissue (i.e., for ages 4–6, muscle tissue [Hg] tended to be higher than the final year’s growth of eye lenses across all systems). Concentrations of Hg in muscle tissue increase as fish age;^49^ hence, the [Hg] in muscle tissues is higher than that in final year of eye lenses in older ages. We suggest further investigations of the dynamics of Hg uptake between eye lens and muscle tissues among different species and ages, to clarify how growth rate affects [Hg] between muscle and eye lenses. In addition, we found concordance in eye lens [Hg] in the final growth year among ages within an ecosystem (Figure S5), suggesting that eye lenses reflect ambient Hg regardless of lens size (a proxy of age). Although Hg in the lens should represent the amount of Hg that accumulated during that year of life, resolving how differences in eye lens volume in innermost versus outermost layers affects Hg concentrations is an area for future study.

From a health risk perspective, fish eyes have been recommended for use in fish oil^50^ and are considered an edible delicacy in some cultures.^50^ Because Hg accumulates in fish eyes at relatively high concentrations and reflects environmental concentrations,^20^ we suggest research on species specific fish eye Hg content may be warranted where they are frequently consumed.

This research is part of a larger study tracking the impacts of hypoxia on fish [Hg] and food web structure. As diet and hypoxia effects are confounding, the future use of otolith microchemistry to assess hypoxia exposure^51^ in fishes can be implemented in combination with our approach to disentangle those effects. In general, studying temporal Hg fingerprints could help to quantify individual exposure to environmental stressors such as hypoxia or other sources of Hg, leading to better assessment and management. Further refinements, for example, analysis of specific isotopic ratios of Hg, could better elucidate sources,^52^ yielding richer details about the chronology of Hg exposure.

## Supporting Information Available

Fish sampling, fish processing, otolith preparation and aging, Hg quality assurance and quality control, external calibration, fish muscle tissue [Hg] as a function of fish total length by ecosystem (Figure S1), Pearson correlation analysis between eye lens radius and otolith length (Figure S2), individual fish variation in Hg and sulfur eye lens profiles (Figure S3), lifetime distribution of annual [Hg] (nanograms per gram of dw) in eye lenses per year of the life of each individual round goby (Figure S4), and a comparison of muscle tissue and final eye lens [Hg] for each age group (Figure S5) (PDF)
