# Supplementary material for: Tracking Fish Lifetime Exposure to Mercury Using Eye Lenses
Source: Environ Sci Technol Lett. 2022 Dec 14;10(3):222–7. doi: 10.1021/acs.estlett.2c00755 (PMC10019466; doi:10.1021/acs.estlett.2c00755)
Supplement: Supplementary file 1 — ez2c00755_si_001.pdf [file ez2c00755_si_001.pdf]

Supporting Information for:

Tracking fish lifetime exposure to mercury using eye lenses

Hadis Miraly[1], N. Roxanna Razavi[1,*], Annabelle A. Vogl[1], Richard T. Kraus[2], Ann Marie Gorman[3], Karin E. Limburg[1,4*]

[1] State University of New York College of Environmental Science and Forestry, Syracuse, NY 13210, USA

[2] U.S. Geological Survey, Great Lakes Science Center, Lake Erie Biological Station, 380 Huron Street, Huron, OH 44839, USA

[3] Ohio Department of Natural Resources, Fairport Fish Research Station, 1190 High St., Fairport Harbor, OH 44077, USA

[4] Department of Aquatic Resources, Swedish University of Agricultural Sciences, Ultuna, Sweden

*Corresponding authors: karin.limburg@slu.se; razavi@esf.edu

Number of Pages: 12

Number of Figures: 5

Number of Tables: 0

Disclaimer: Any use of trade, product, or firm names is for descriptive purposes only and does not imply endorsement by the U.S. Government.

Supplementary Materials and Methods

Fish sampling

Round Goby from the Baltic Sea (n = 28) were collected in August and October 2020 using gill nets from two sites: offshore of the northern coastal city of Gävle, Sweden (n = 10), and an area near Muskö in the southern part of the Stockholm archipelago (n = 18). The sampling was performed by the Department of Aquatic Resources, Swedish University of Agricultural Sciences.

Round Goby from Lake Erie (n = 71) were collected in June and September 2019 using bottom trawls in the Western Basin (n = 33) as well as the Central Basin (n = 38). Differences in age structure among ecosystems affected the average age and lengths of Round Goby collected. Lake Erie had the youngest fish (Ages 0 – 3), followed by the St. Lawrence River (Ages 0 – 5), and the Baltic Sea (Ages 1 – 6, no Age 2 individuals). Average lengths (± s.d.) ranged from 158 ± 27, 87 ± 21, 153 ± 57 in the Baltic Sea, Lake Erie, and the St. Lawrence River, respectively. The USGS Great Lakes Science Center and the Ohio Department of Natural Resources (Ohio DNR) collected fish from the Western and Central Basins, respectively. Round Goby in the St. Lawrence River were collected in June and July 2020 using standardized nearshore seining in the Thousand Islands archipelago, located at the outflow of Lake Ontario in the upper St. Lawrence River (n = 28). The sampling was performed by fisheries technicians at the Thousand Islands Biological Station. All field sampling was carried out according to American Fisheries Society guidelines for the care and use of fish,[1] and approved procedures of the respective agencies. All fish were frozen at −20°C until dissected.

Fish processing

Fish were measured for total length and weight and were dissected for eye lenses, otoliths, and muscle tissue samples. Otoliths were extracted, rinsed, cleaned in 50% bleach solution, and rinsed again to remove any adhering tissue. Otoliths were then air-dried and stored in microcentrifuge tubes for later examination. Eye lenses in each fish were removed, cleaned of adhering macular tissue, air-dried, and stored in microcentrifuge tubes. Because fish were analyzed for Hg in muscle tissue, dissection tools and

trays were all made of stainless steel and new scalpel blades were used for each fish dissected to prevent contamination. For fish > 60 mm, dorsal muscle tissue with no skin and bone was removed, frozen and ~ 5 g of fillet of each individual was freeze-dried for 24 hours. All Lake Erie fish were analyzed as whole fish, and four St. Lawrence River individuals < 60 mm were processed whole for Hg content. Whole body Hg concentrations were converted to muscle Hg concentrations following Peterson et al.[2] as mentioned in-text. The Baltic Sea fish muscle samples were oven-dried at 45 °C overnight prior to being transported, then freeze-dried at SUNY-ESF for 24 hours.

Eye lenses were air dried and assumed to be completely dry prior to analysis on the LA-ICPMS. All eye lenses were embedded in epoxy (Struers EpoFix) and then sectioned through the core with a Buehler IsoMet cutting machine.[3] Each section was polished by hand with polishing paper from P800 down to a grit size P2500 until the eye lens core became visible using a Zeiss Axio compound microscope at 10X magnification. The cross-sections were placed on petrographic glass slides with double-stick tape for microchemistry analyses.

Otolith preparation and ageing

Otoliths were embedded in epoxy and then were polished by hand with polishing paper ~~from~~ P400 down to a grit size of P2500 until the annual rings became visible using a dissecting microscope. Fish age was determined by counting the growth bands (annuli) on thin sections of otoliths twice,[4] then otoliths were photographed with an OLYMPUS dissecting microscope at 20X magnification to make measurements between each annulus from the core. Additional ageing of otoliths was completed by a second reader for all of the samples (percent agreement = 86 %, mean coefficient of variation = 11%).

Hg quality assurance/quality control

Strict quality control measures were followed including duplicates of all samples (average relative percent difference (RPD) = 6.6 %, n = 330), matrix spikes/ matrix duplicates, method blanks. Certified standard reference materials, TORT-3 (Lobster hepatopancreas; National Research Council Canada) and DORM-4 (Fish protein homogenate; National Research Council Canada) were analyzed

every 5 sample runs. All recoveries averaged within 10% of reference values (TORT-3 recovery = 104 ± 2 %, n = 9; DORM-4 recovery = 100 ± 4 %, n = 61).

External calibration

To tune the LA-ICPMS, the National Institute of Standards and Technology 612 (NIST 612) standard reference glass was used. Since eye lenses are a protein rich structure, an in-house standard composed of fish protein certified reference material (DORM-4) was used. The DORM-4, as mentioned above, was used for quality control purposes to measure the total Hg content of muscle and whole body tissues. It consists of 18 gr of fish protein. Therefore, we developed this procedure in the lab to correct for instrument drift and to convert raw intensities to mass concentrations. Transects were set to pass through the core and traverse the eye lens completely. Analysis was completed in single quadrupole mode to collect data on the selected trace element (Hg). A metric of analytical precision for ^{202}Hg, the most abundant Hg stable isotope, is the average relative standard deviation (RSD) for DORM-4 (n = 30), was 14.9 % across all runs.

Figure S1. Fish muscle tissues [Hg] as a function of fish total length (mm) by ecosystem; weak relationships were found. Note, data shown here for Lake Erie and the four smallest (< 60 cm) individuals for the St. Lawrence River were analyzed as whole fish. Models in-text were based on converted concentrations from whole to muscle tissue following Peterson et al.[2]

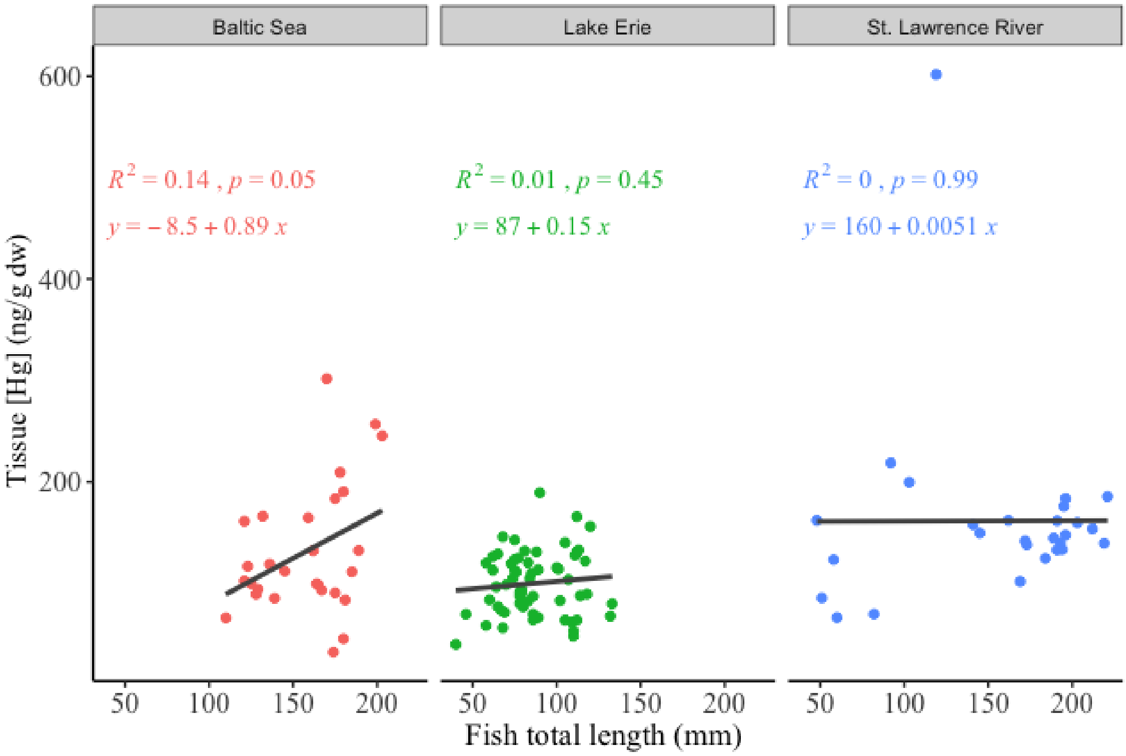

Figure S2. Pearson correlation analysis between eye lens radius and otolith length, with a 95 % confidence interval shown in grey. Eye lens radius and otolith length were strongly correlated for all ecosystems (Pearson correlation, n = 114, r = 0.82, $p < 0.05$), as well as individually (regressions not shown; the Baltic Sea (r = 0.68, n = 28, $p < 0.05$), Lake Erie (r = 0.6, n = 60, $p < 0.05$), and for the St. Lawrence River (r = 0.77, n = 26, $p < 0.05$)).

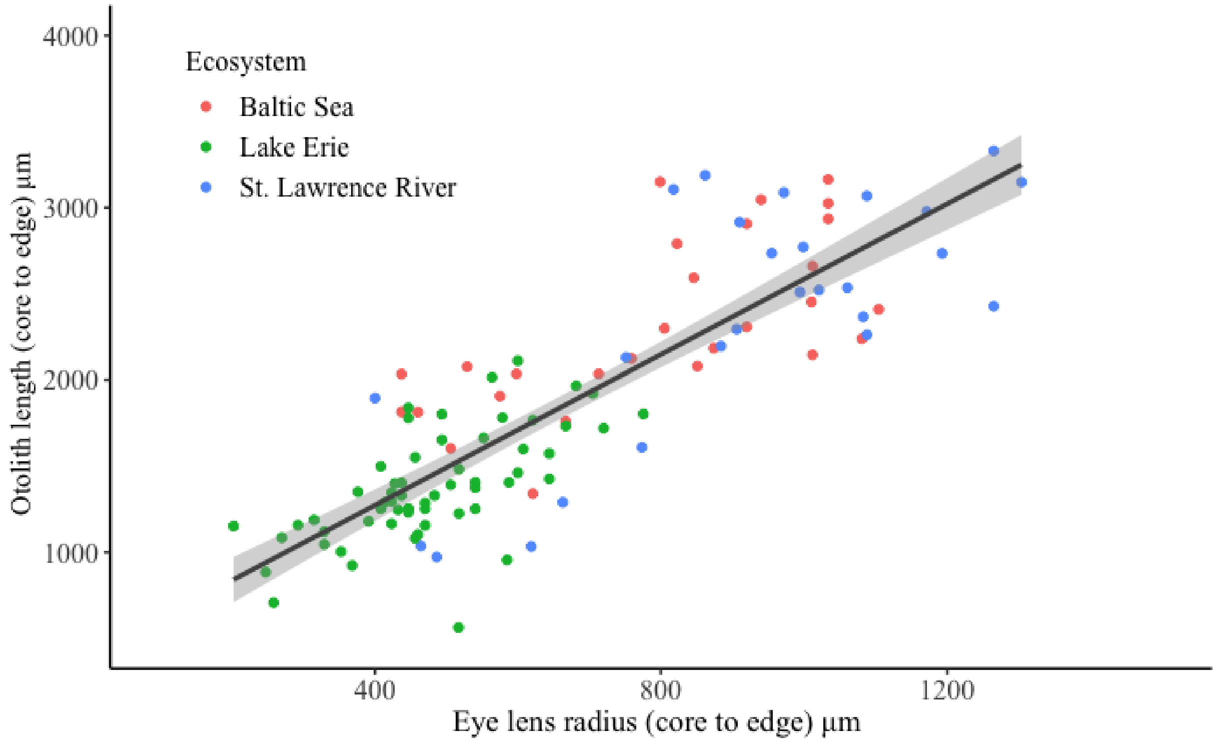

Figure S3. Individual fish variation in Hg and sulfur eye lens profiles. Mercury profiles (top row) shown for individuals with a loess fit. Note here that grey lines are individuals from a single age group for each ecosystem. The colored line is a loess fit. Sulfur profiles (bottom row) are from the same individuals displayed in the Hg profiles.

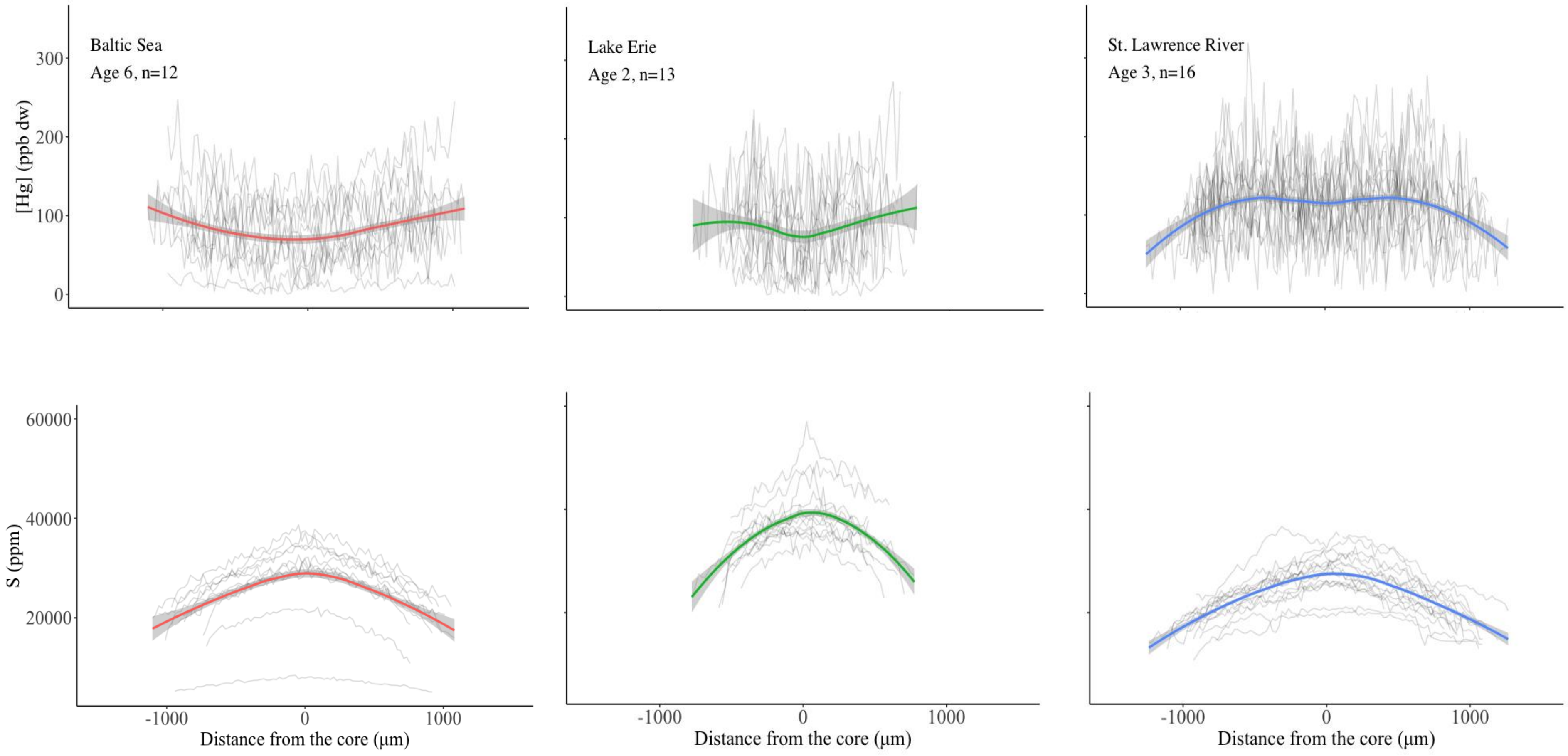

Figure S4. Density plot demonstrating all age-based eye lens Hg measurements in individual fish (e.g., an Age 3 individual would have 4 data points that represent Hg concentration for each year of its life). Measured annual eye lens [Hg] increases with age in Lake Erie and Baltic Sea but declines with age in the St. Lawrence River. Here we show annual [Hg] (ng/g dw) in eye lenses per year of life of each individual Round Goby for (A) the Baltic Sea, n = 28, (B) Lake Erie, n = 71, and (C) the St. Lawrence River, n = 28. Pairwise comparisons showed that eye lens [Hg] between the Baltic Sea and the St. Lawrence River were significantly different for age 0 (t (170) = -4.869, $p < 0.05$), age 1 (t (172) = -4.059, $p < 0.05$), and age 2 (t (183) = -3.759, $p < 0.05$). In all cases the St. Lawrence River was significantly higher compared to the Baltic Sea. The St. Lawrence River was significantly higher for age 0 compared to Lake Erie (t (171) = -4.741, $p < 0.05$).

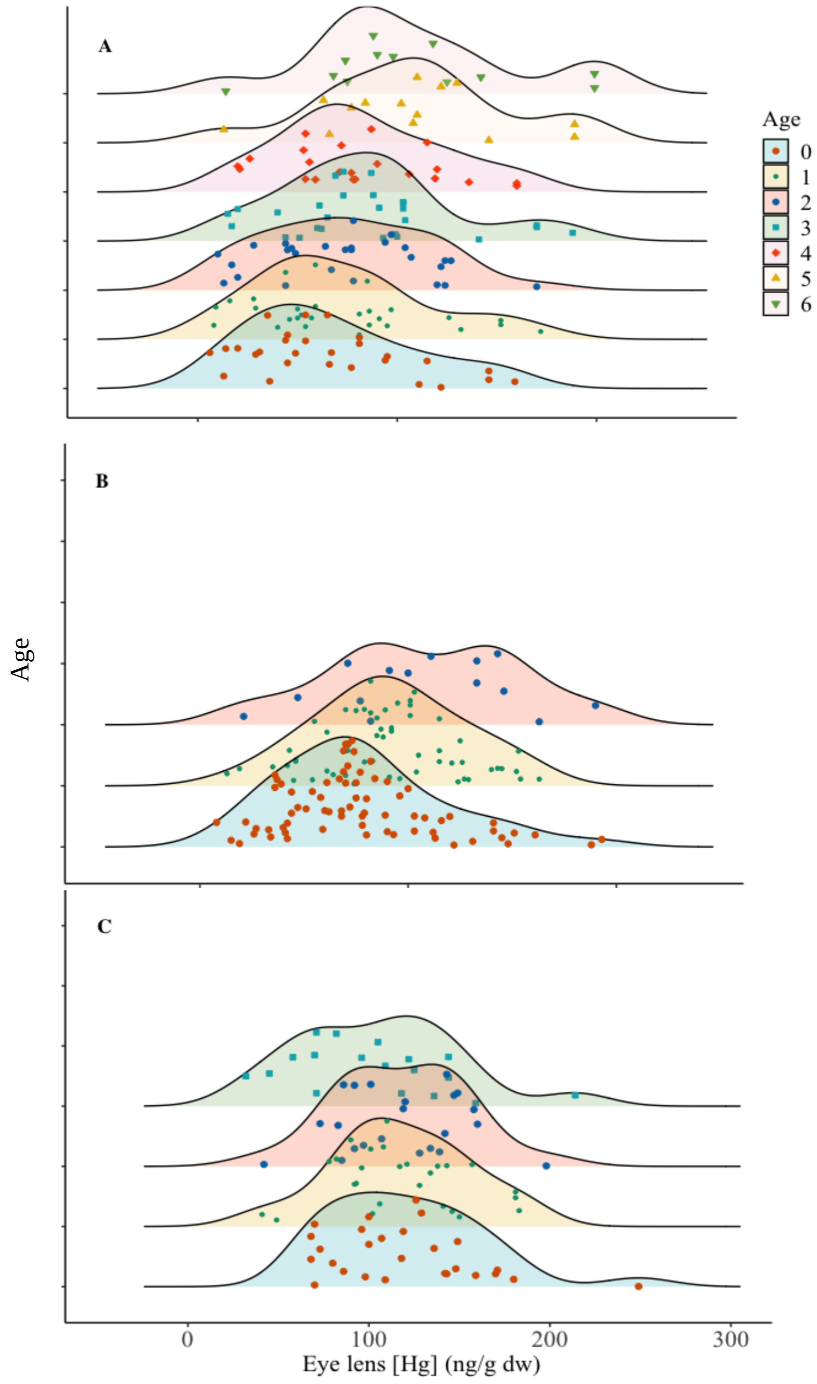

A
B
C
Age
0
1
2
3
4
5
6
Age
0
100
200
300
Eye lens [Hg] (ng/g dw)

Figure S5. Comparison of muscle tissues and measured eye lens [Hg] in the final year for each age group in (A) the Baltic Sea, (B) Lake Erie, and (C) the St. Lawrence River. Significant differences between tissue types are indicated by an asterisk (Tukey HSD, $p < 0.05$). Note, not all age groups Round Goby were collected from each ecosystem. In contrast, eye lenses provide a continuous record of Hg exposure in an individual's lifetime (see Figure S4) and do not require sampling of all age classes to provide age specific exposures as with traditional sampling. In addition, within an ecosystem, we did not find significant differences among ages in the final year of eye lens [Hg] (ANOVA, $p > 0.05$).

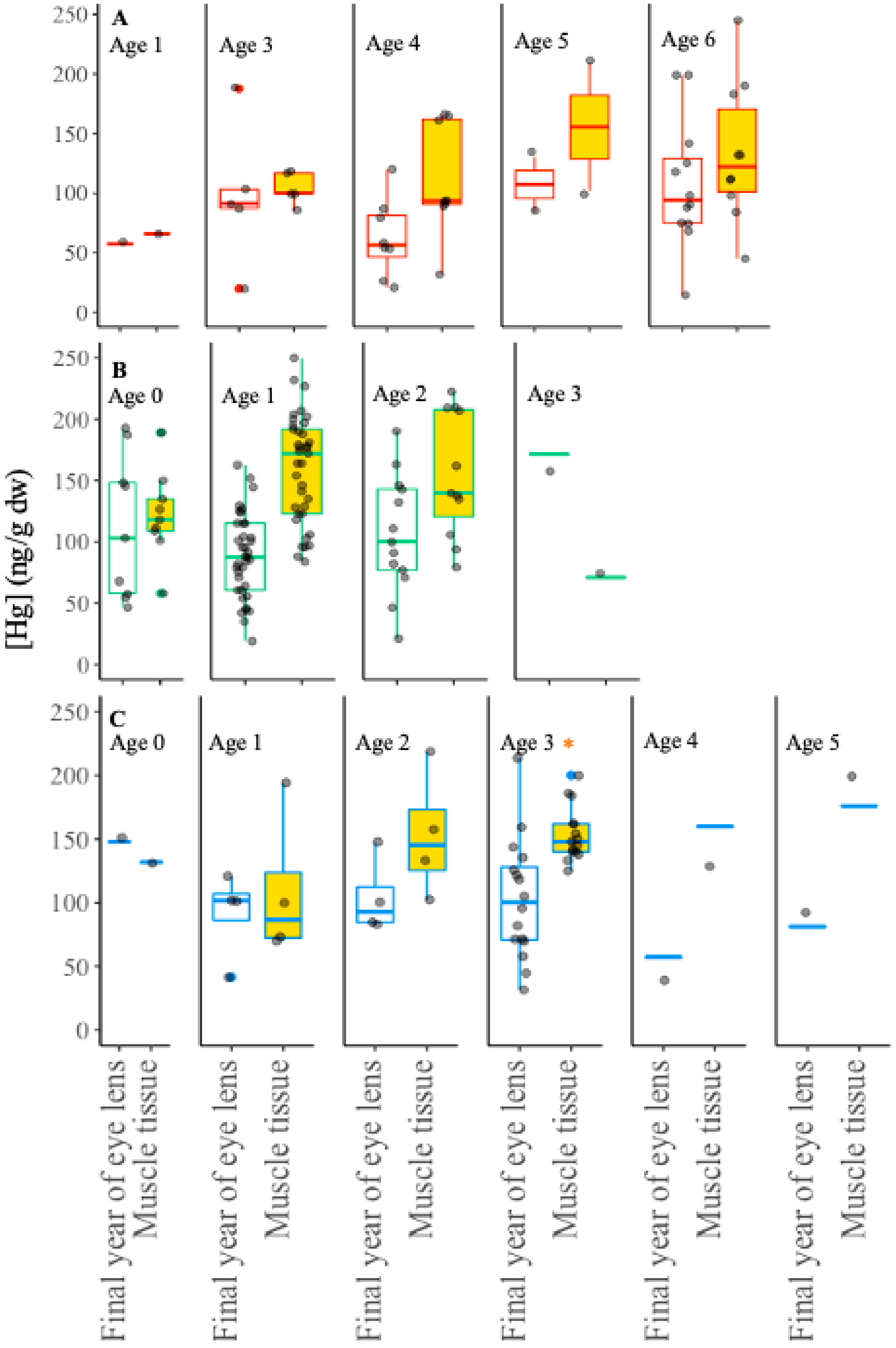
A
Age 1
Age 3
Age 4
Age 5
Age 6
B
Age 0
Age 1
Age 2
Age 3
C
Age 0
Age 1
Age 2
Age 3 *
Age 4
Age 5
[Hg] (ng/g dw)
0
50
100
150
200
250
Final year of eye lens
Muscle tissue

References Cited

(1) Jenkins, J. A.; Jr, H. L. B.; Bowker, J. D.; Bowser, P. R.; Macmillan, R.; Nickum, J. G.; Rachlin, J. W.; Rose, J. D.; Sorensen, W.; Warkentine, B. E.; Whitledge, G. W.; Jenkins, J. A.; Jr, H. L. B.; Bowker, J. D.; Paul, R.; Macmillan, J. R.; Nickum, J. G.; Rachlin, J. W.; Rose, J. D.; Sorensen, W.; Warkentine, B. E.; Whitledge, G. W.; Bart, H. L.; Bowser, P. R.; Macmillan, J. R.; Nickum, J. G.; Rachlin, J. W.; Rose, J. D.; Sorensen, P. W. Guidelines for Use of Fishes in Research—Revised and Expanded, 2014. *Fisheries* **2014**, *39* (9), 415–416.

(2) Peterson, S. A.; Van Sickle, J.; Herlihy, A. T.; Hughes, R. M. Mercury concentrations in fish from streams and rivers throughout the western United States. *Environ. Sci. Technol*. **2007**, *41*(1), 58–65.

(3) Stounberg, J.; Thomsen, T. B.; Heredia, B. D.; Hüssy, K. Eyes and ears: a comparative approach linking the chemical composition of cod otoliths and eye lenses. *J. Fish Biol*. **2022**, *101* (4), 985-995.

(4) Huo, B.; Madenjian, C. P.; Xie, C. X.; Zhao, Y.; Brien, T. P. O.; Czesny, S. J. Age and growth of Round Gobies in Lake Michigan, with preliminary mortality estimation. *J. Great Lakes Res.* **2014**, *40* (3), 712–720.
